# Organozinc Precursor-Derived Crystalline ZnO Nanoparticles: Synthesis, Characterization and Their Spectroscopic Properties

**DOI:** 10.3390/nano8010022

**Published:** 2018-01-04

**Authors:** Yucang Liang, Susanne Wicker, Xiao Wang, Egil Severin Erichsen, Feng Fu

**Affiliations:** 1 Institut für Anorganische Chemie, Eberhard Karls Universität Tübingen, Auf der Morgenstelle 18, 72076 Tübingen, Germany; susanne.wicker@ipc.uni-tuebingen.de; 2 School of Physics and Electronics, Hunan University, Changsha 410082, China; xiao_wang@hnu.edu.cn; 3 Laboratory for Electron Microscopy, University of Bergen, Allégaten 41, 5007 Bergen, Norway; Egil.Erichsen@mnfa.uib.no; 4 College of Chemistry & Chemical Engineering, Yan’an University, Shaanxi Key Laboratory of Chemical Reaction Engineering, Yan’an 716000, China

**Keywords:** organozinc precursor, thermal decomposition, zinc oxide, nanoparticle, size effect, spectroscopic properties

## Abstract

Crystalline ZnO_-ROH_ and ZnO_-OR_ (R = Me, Et, *i*Pr, *n*Bu) nanoparticles (NPs) have been successfully synthesized by the thermal decomposition of in-situ-formed organozinc complexes Zn(OR)_2_ deriving from the reaction of Zn[N(SiMe_3_)_2_]_2_ with ROH and of the freshly prepared Zn(OR)_2_ under an identical condition, respectively. With increasing carbon chain length of alkyl alcohol, the thermal decomposition temperature and dispersibility of in-situ-formed intermediate zinc alkoxides in oleylamine markedly influenced the particle sizes of ZnO_-ROH_ and its shape (sphere, plate-like aggregations), while a strong diffraction peak-broadening effect is observed with decreasing particle size. For ZnO_-OR_ NPs, different particle sizes and various morphologies (hollow sphere or cuboid-like rod, solid sphere) are also observed. As a comparison, the calcination of the fresh-prepared Zn(OR)_2_ generated ZnO_-R_ NPs possessing the particle sizes of 5.4~34.1 nm. All crystalline ZnO nanoparticles are characterized using X-ray diffraction analysis, electron microscopy and solid-state ^1^H and ^13^C nuclear magnetic resonance (NMR) spectroscopy. The size effect caused by confinement of electrons’ movement and the defect centres caused by unpaired electrons on oxygen vacancies or ionized impurity heteroatoms in the crystal lattices are monitored by UV-visible spectroscopy, electron paramagnetic resonance (EPR) and photoluminescent (PL) spectroscopy, respectively. Based on the types of defects determined by EPR signals and correspondingly defect-induced probably appeared PL peak position compared to actual obtained PL spectra, we find that it is difficult to establish a direct relationship between defect types and PL peak position, revealing the complication of the formation of defect types and photoluminescence properties.

## 1. Introduction

Nanostructured metal oxide nanoparticles have attracted increasing attention due to their specific physical and chemical properties in optic, magnetism, conductivity and reactivity. A lots of metal oxides have been applied to industrial products in sensors, in cosmetics, in medical diagnosis, or as new devices for optical and electronic applications. These metal oxides were often prepared by top down from physical approach and bottom up from chemical method. The physical method is very difficult to control uniform shape and obtain very small grain size less than 10 nm [1]. Nowadays, chemical methods (such as co-precipitation, microemulsion, solvothermal synthesis, thermal decomposition, sol-gel technique, sonochemical route, microwave-assisted technique and electrochemical deposition) are becoming a popular strategy for fabricating nanostructured metal oxide or metal hydroxide with controllable morphology and uniform particle size at molecular level. Among these metal oxides (SnO_2_, ZnO, NiO, TiO_2_, Fe*_x_*O*_y_*, Co*_x_*O*_y_*, In_2_O_3_, WO_3_, CoFe_2_O_4_ etc.) [2,3,4,5,6,7,8,9,10,11], nanostructured ZnO materials were extensively investigated due to several advantages in friendly environment, high electron mobility, flexible synthesis methods, various morphologies, the most interesting hot research targets for building field-effect transistors and energy harvesting (piezoelectric nanogenerators and photovoltaics) [12,13,14,15,16,17], for bioimaging and drug delivery [18] and sensors [1,19]. 

Zinc oxide with a large direct wide band gap of about 3.37 eV at room temperature and a large exciton binding energy of 60 meV [20,21] has attracted significant attention because of its special electronic and photonic properties and its broad applications in electronics, optoelectronics, electrochemistry, fabricating piezoelectric nanodevices, light emitting diodes, solar cell, nanolasers, sensors and catalysis [22]. More recently, transition-metal-doped p-ZnO NPs-based sensory array can be used for instant discrimination of explosive vapours [23]. Up to now, 1D nanostructured ZnO materials have been prepared by using various synthetic approaches including wet chemical method [22,24,25,26], physical or chemical vapour deposition [22,27], pulsed electro-chemical deposition [22,28,29,30,31,32], pulsed laser deposition [33,34], molecular beam epitaxy [35], electrospinning [36] and microwave heating technique [37,38,39,40]. Wet chemical approach is a facile, cost-effective and convenient method using cheap inorganic zinc salts as precursors in an alkaline medium to fabricate nanostructured ZnO materials with uniform morphologies such as nanospheres, nanotubes, nanorods, nanoprisms, nanobelts and nanowires and pure or transition-metal-doped ultrathin ZnO nanosheets [22,23,24,25,26]. For example, the monodisperse morphology-controlled ZnO troughs could be prepared at the air-water interface under mild conditions [41] and ZnO microspheres could be fabricated by the chemical conversion of ZnSe [42]. But its shortage is that the ZnO nanoparticles obtained often show polydispersity or poor uniformity or aggregations in solution. To overcome these shortcomings, a thermal decomposition method was developed in order to control the dispersibility or hinder self-assemble of single nanoparticle in the presence of stabilizer [40,43]. The formation mechanism of ZnO nanocrystals can be monitored by in-situ IR spectra [44,45]. Moreover, the improvement of the dispersibility of ZnO NPs can also be carried out by microemulsion method [46]. However, in general, thermal decomposition of inorganic zinc salts such as ZnC_2_O_4_∙2H_2_O, Zn_5_(OH)_6_(CO_3_)_2_, Zn(CH_3_COO)_2_∙2H_2_O and Zn_3_(OH)_4_(NO_3_)_2_ often occurs at a high temperature (300–600 °C) with a long reaction time to generate crystalline ZnO NPs [47,48]. Compared to inorganic zinc salts, organozinc complexes as a potential candidate can readily decompose at a low temperature. For the past ten years, some organozinc complexes have been chosen as the zinc source for the preparation of nanostructured zinc oxide or porous zinc oxide materials. In these complexes, reaction temperature and the use of different organozinc precursors markedly influenced the quality and properties of the obtained nanostructured ZnO, such as mono/polydispersibility, nucleation and growth rate and optical properties. For example, the thermal decomposition of diethylzinc under O_2_-rich environment produced würtzite ZnO nanocrystals via hot-injection method in the presence of trioctylphosphine oxide or alkylamines [49]. The alcoholization of diethylzinc generated zinc alkoxide, which could be hydrolysed to form crystalline zinc oxide with a particle size of 3–5 nm and the resulting ZnO particles could further aggregate together to form spherical particles that have a large surface area and enhanced reactivity [50]. Metal zinc NPs prepared by bis(cyclohexyl) zinc complex in the presence of different solvents and stabilizers under argon protection were exposed to moisture air to be slowly oxidized and form size and shape-controlled crystalline ZnO NPs [51,52].

In 1994, the heteroleptic zinc complexes MeZn(O*i*Pr) or MeZn(O*t*Bu) were used as a single precursor for growth of ZnO film by metal-organic chemical vapour deposition(CVD) [53]. In 2005, Driess and co-workers further investigated the CVD process of heterocubane precursor [MeZn(O*i*Pr)]_4_ for the formation of size-selected ZnO NPs and proposed the reaction mechanism of the gas-phase decomposition of [MeZn(O*i*Pr)]_4_ [54,55]. Moreover, the pyrolysis of EtZnO*i*Pr could also form monodisperse spherical ZnO NPs with an average size of 3.1 ± 0.3 nm in the presence of trioctylphosphine oxide; the resulting ZnO NPs indicated a blue-shifted phenomenon of excitonic absorption peak, revealing a quantum confinement effect of nanostructured ZnO [56]. In 2007, Polarz and co-workers explored in detail the reaction of ZnMe_2_ with polyethylene glycol in toluene and the formed [MeZnOPEG_400_] gels could be used as a ZnO precursor for the preparation of mesoporous ZnO materials [57]. Except for the above-mentioned, these organozinc complexes—[MeZn(OCH_2_CH_2_OMe)] and RZn(OH)-type such as [*t*BuZn(μ-OH)]*_n_* and its derivatives—could also be converted into ZnO NPs [58,59,60]. In addition to these zinc complexes, the directly thermal decomposition of zinc-organic framework Zn_4_O(BDC)_3_ (MOF-5, BDC represents benzene-1,4-dicarboxylate) at above 400 °C formed amorphous carbon-covered ZnO NPs [61]. Decomposition of zinc acetylacetonate in oleylamine yielded ZnO NPs of 7–10 nm with increasing reaction temperature [62]. Furthermore, the doping or surface modification of heterometals can also effectively adjust the electrical, optical and magnetic properties of obtained ZnO materials.

In addition, the photoluminescent property of bulk or nanostructured zinc oxide particles were also extensively investigated. Especially, zinc oxide materials with different morphologies showed a variety of optical properties. ZnO nanowires synthesized with a vapour phase transport process via catalysed epitaxial crystal growth on the substrate indicated a band gap at 377 nm (3.29 eV) [63]. ZnO nanowires prepared by vapour transport had a strong emission at 380 nm (3.26 eV) [64]. ZnO materials with spheres, triangular prisms and rods prepared by thermal decomposition method displayed various UV emission ranging from 3.19 eV (spheres) to 3.30 eV (triangular prisms), implying that the band gaps depended on the morphology of nanostructured materials [65]. Hydrothermally synthesized ZnO nanorods had a UV emission at 390 nm, a broad shoulder (400–425 nm) and weak peaks at 417, 446 and 465 nm from the photoluminescent spectrum [24]. For ZnO quantum rods, different photoluminescent properties were also observed due to quantum confinement effects [66]. However, the photoluminescence spectrum of ZnO prepared by physical method such as thermal evaporation deposition showed different emission at various temperatures, for example, bound exciton (3.354 eV), free excitons (3.375 and 3.421 eV), the first/second/third longitudinal optical phonon order replicas (3.315/3.243/3.171 eV) of free exciton (3.375 eV) and donor acceptor pairs (3.188 eV) at low temperature (6 K), the first longitudinal optical phonon replica (3.315 eV) of free exciton at room temperature [67]. The photoluminescence characteristics of catalyst free ZnO nanowires at different temperatures and excitation intensities were explored by Mohanta and Thareja [68]. Moreover, ZnO NPs in vapour phase showed 42 meV shift in peak position of PL spectrum compared to that of bulk ZnO [69]. As an extensive research the exciton-exciton scattering in vapour phase ZnO NPs was also investigated [70]. In addition, CdO-modified ZnO tailored the band gap of ZnO to achieve luminescence from ultraviolet to the blue and green spectral region [71]. With increasing Cd concentration in ZnCdO, the band gap gradually decreased due to a larger ionic radius of Cd^2+^ [72]. Note that the photoluminescence spectrum of ZnCdO with 50 wt % Cd showed an abnormal red-blue-redshift with increasing temperature [73]. Based on these investigations, it is easier to find that the photoluminescent behaviour of nanostructured ZnO materials depends on size, morphology, surface defect types and doping of surface impurity as well as preparation methods.

In this study, we investigated in detail the thermal decomposition of the in-situ formed zinc alkoxide, Zn(OR)_2_ [74] (R = Me, Et, *i*Pr and *n*Bu), which originated from the reaction between Zn[N(SiMe_3_)_2_]_2_ [75] and alkyl alcohol (MeOH, EtOH, *i*PrOH and *n*BuOH) in the presence of oleylamine, to fabricate the nanostructured ZnO particles and explored the influence of the type of alkyl alcohol, reaction temperature and the capped-stabilizer on morphology, size, the degree of crystallinity, spin paramagnetic resonance spectra and optical property of ZnO NPs obtained. As a comparison, the direct pyrolysis of zinc alkoxide was also investigated in the presence or absence of high boiling-point solvent oleylamine or a mixture of oleic acid and 1-octadecene.

## 2. Results and Discussion

### 2.1. Structural Characterization of a Series of Nano-Structured Zinc Oxide Particles ZnO_-ROH_, ZnO_-OR_ and ZnO_-R_ (R = Me, Et, iPr, nBu, Gc)

**Scheme 1:**
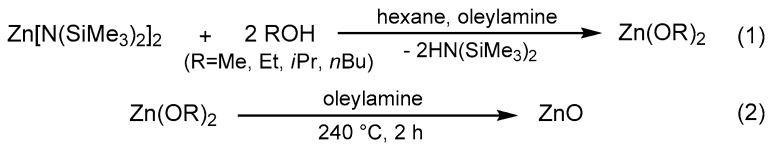
Proposed reaction process for the formation of nanostructured ZnO particles using homoleptic zinc silylamido complex Zn[N(SiMe_3_)_2_]_2_ as a zinc precursor and alkyl alcohol as a reactant in the presence of high boiling-point solvent as a stabilizer.

Homoleptic organozinc complexes including alkyl zinc (ZnMe_2_ or Zn(C_6_H_11_)_2_ etc.) and zinc amide complexes (Zn[N*i*Pr_2_]_2_ or Zn[N(SiMe_3_)_2_]_2_ etc.) have been used as a zinc precursor for the preparation of metal zinc NPs due to low thermodynamic stability of Zn–N and Zn–C bonding [51,52]. But the direct decomposition of these complexes cannot result in the formation of zinc oxide NPs due to the absence of oxygen source. In present work, ZnO_-ROH_ NPs were prepared via thermal decomposition of an in-situ formed zinc alkoxide which derived from the reaction of Zn[N(SiMe_3_)_2_]_2_ with alkyl alcohol ROH (R = Me, Et, *i*Pr and *n*Bu) in the presence of high boiling-point solvent oleylamine. Under an identically synthetic condition, reaction procedure is proposed as follows:

As viewed in Scheme 1, zinc silylamido complex first reacts with alkyl alcohol to form zinc alkoxide complex and eliminate silylamide ligand [74], follows by in-situ thermal decomposition to generate ZnO NPs. The thermal decomposition temperature mainly depends on the nature of formed intermediate zinc alkoxide. Moreover, volatile compositions including by-product and unreacted alkyl alcohol can be removed completely under vacuum.

#### 2.1.1. Crystalline ZnO_-MeOH_ NPs

**Figure 1:**
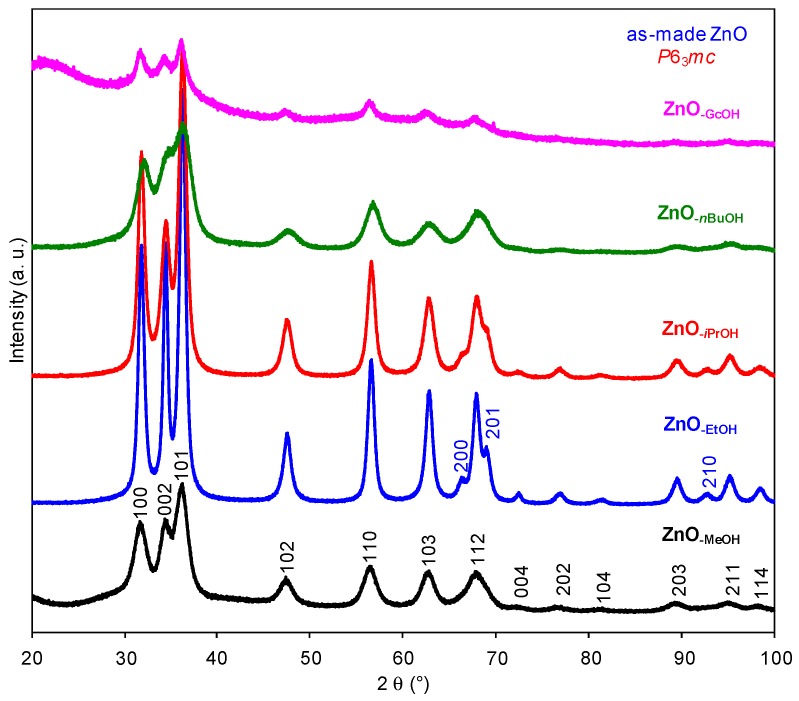
Wide-angle PXRD patterns of as-synthesized ZnO_-ROH_ NPs by using Zn[N(SiMe_3_)_2_]_2_ as a zinc precursor and alkyl alcohol as a reactant and solvent in the presence of stabilizer.

**Figure 2:**
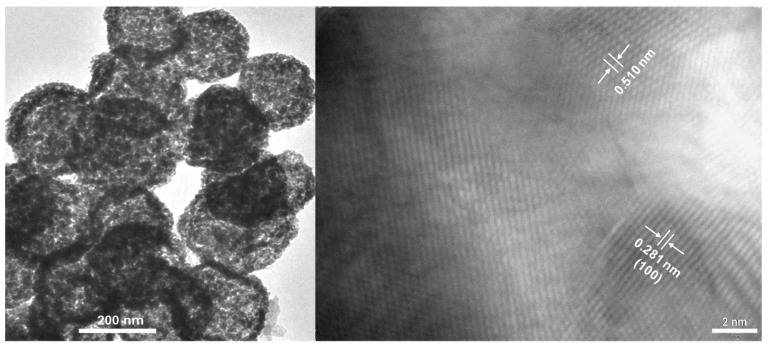
(High-resolution) TEM images of the crystalline ZnO_-MeOH_ NPs with visual lattice fringes (correspond to crystal planes).

According to synthetic procedure (Scheme 1), when methanol was used as a reactant and solvent in the presence of oleylamine, ZnO_-MeOH_ particles were obtained. The powder X-ray diffraction (PXRD) pattern of ZnO_-MeOH_ clearly shows several well-resolution diffraction peaks, which can be indexed as (100), (002), (101), (102), (110), (103) and (112) reflections (Figure 1), revealing a characteristic würtzite structure of ZnO (a = 3.25 Å, c = 5.21 Å, *P6_3_mc*, Powder Diffraction File Database (PDF-2), entry: JCPDS 36-1451). For the synthesis of ZnO_-MeOH_ NPs, we observed that the dropwise addition of hexane solution of Zn[N(SiMe_3_)_2_]_2_ into reaction system quickly formed the sphere-shaped aggregations. Part of the aggregations was separated, washed several times with methanol under argon protection and dried under vacuum to get white powder. Elemental analysis confirmed that the white powder was Zn(OMe)_2_ (found wt % C: 19.38, H: 4.39, N: 0.09; calcd. C: 18.84, H: 4.74). IR spectrum of white powder clearly shows characteristic C–O vibration at 1050 cm^−1^, C–H stretching vibrations in the range of 2820~2930 cm^−1^ and bending vibration at 1450 cm^−1^ from –CH_3_ group and Zn–O vibration at 482 and 560 cm^−1^ (Figure S1, Electronic Supplementary Information (ESI)), further verifying the formation of pure intermediate Zn(OMe)_2_. It is noted that the sphere-shaped aggregations {Zn(OMe)_2_}*_n_* after removal of volatile compositions could not effectively disperse in oleylamine solvent even if it was heated to 240 °C. The thermal decomposition of in-situ formed {Zn(OMe)_2_}*_n_* generated ZnO_-MeOH_ NPs still preserved sphere-like aggregations with average particle size of 234.5 ± 6.1 nm (Figure 2, left). These agglomerations consist of small nanoparticles with an average particle size of 5.9 ± 0.3 nm and the lattice fringes are clearly visible with spacing of 0.281 and 0.510 nm and the lattice spacing of 0.281 nm corresponds to (100) planes of ZnO with *P6*_3_*mc* symmetry (Figure 2, right). Moreover, the visual grain boundaries clearly confirm the connection of small particles each other. In addition, according to the Scherrer formula, *D*_hkl_ = *K*λ/(βcosθ), where *D*_hkl_ is the mean size of the ordered crystalline grain size, *K* is a dimensionless shape factor (0.89), λ is the X-ray wavelength (0.15406 nm), β is the full width at half the maximum intensity (in radians), θ is the Bragg angle (in degrees), the calculated mean particle size of ZnO_-MeOH_ is 4.6 nm from the (100) plane and 4.9 nm from the (101) reflection plane. These results are in good agreement with the result measured by transmission electron microscopy (TEM) images. Detailed particle sizes determined by different methods are listed in Table 1.

#### 2.1.2. Alkyl Alcohol Effect

When EtOH, *i*PrOH and *n*BuOH were respectively used to replace MeOH in the preparation of ZnO_-MeOH_, the corresponding nanoscaled ZnO_-ROH_ (R = Et, *i*Pr, *n*Bu) particles were obtained. All ZnO_-ROH_ NPs have a same crystal structure (space group: *P6*_3_*mc*) verified by PXRD patterns (Figure 1) as that of ZnO_-MeOH_, but the ZnO_-EtOH_ and ZnO_-*i*PrOH_ show a relatively narrow and strong diffraction peaks compared to that of ZnO_-MeOH_ and ZnO_-*n*BuOH_, implying that use of different alkyl alcohols as a reactant and a reaction solvent can effectively influences the crystalline particle size of zinc oxides obtained and the results show a strong diffraction peak-broadening/weakening effect with decreased grain size [76]. This phenomenon is probably caused by the change of growth rate of ZnO particles influencing by decomposition temperature, dispersibility and solubility of formed intermediate Zn(OR)_2_ in oleylamine during the thermal decomposition.

**Figure 3:**
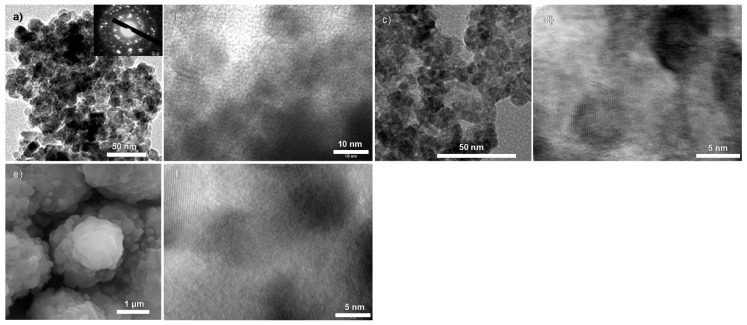
(High-resolution) TEM images of crystalline (**a**,**b**) ZnO_-EtOH_ and (**c**,**d**) ZnO_-*i*PrOH_, and SEM and TEM image of (**e**,**f**) ZnO_-*n*BuOH_. The inset in (**a**) is the SAED pattern.

Moreover, for ZnO_-EtOH_ NPs, TEM images and the selected area electron diffraction (SAED) pattern clearly revealed a crystalline structure with *P6*_3_*m*c symmetry (Figure 3a,b). For the sphere-like ZnO_-*i*PrOH_ and plate-like-aggregated ZnO_-*n*BuOH_ particles (Figure 3c,e), the lattice fringes can also be observed (Figure 3d,f), although ZnO_-*n*BuOH_ has a series of slightly broad and weak diffraction peaks (similar to that of ZnO_-MeOH_). The particle size calculated by Scherrer formula is listed in Table 1: 10.7 nm from the (100) plane and 10.2 nm from the (101) reflection, respectively, for the spherical ZnO_-EtOH_; 9.0 nm from the (100) plane and 8.3 nm from the (101) reflection plane, respectively, for sphere-like ZnO_-*i*PrOH_; 4.3 nm from (100) plane and 4.0 nm from the (101) reflection plane, respectively, for ZnO_-*n*BuOH_. These results are quite close to corresponding average particle size measured by TEM images (Table 1, 11.7 ± 0.6 nm for ZnO_-EtOH_; 7.5 ± 0.2 nm for ZnO_-*i*PrOH_; for the multiply plate-like-aggregated ZnO_-*n*BuOH_ spheres (Figure 3e), it is very difficult to accurately measure their particle sizes). Based on these results, it is obvious to observe size-induced the weakening of diffraction peak intensity and the broadening of diffraction peaks (grain size: ZnO_-EtOH_ > ZnO_-*i*PrOH_ > ZnO_-MeOH_ > ZnO_-*n*BuOH_) and alkyl alcohol-induced the change of morphologies. As a special example, when high boiling-point glycerol (GcOH) was used as a reactant, the formed intermediate zinc glycerolate Zn[OCH_2_CH(OH)CH_2_O] [77,78,79] (monoclinic *P2*_1_/*c*, Powder Diffraction File Database (PDF-2), entry: JCPDS 23-1975) could not be thermally decomposed at 240 °C for 2 h under an identical condition to yield crystalline ZnO_-GcOH_ NPs. However, ZnO_-GcOH_ NPs can be prepared at 320 °C for 2 h in the presence of oleic acid and 1-octadecene. Obviously, this thermal decomposition temperature is markedly less than 400–500 °C which has been previously used for the preparation of ZnO NPs by choosing zinc glycerolate as a molecular precursor [77,79]. Compared to ZnO_-ROH_ (R = Me, Et, *i*Pr and *n*Bu), several weak diffraction peaks are observed for ZnO_-GcOH_ (Figure 1). The particle size calculated by Scherrer formula is 3.8 nm from the (100) plane and 3.6 nm from the (101) reflection plane, respectively.

As a comparison, thermal decomposition of intermediate Zn(O*i*Pr)_2_ forms ZnO NPs composing of spherical agglomerations of crystallites. This result is quite similar to small-sized ZnO prepared previously via a hydrolysis of Zn(O*i*Pr)_2_ [50]. In the present research, it is very difficult to perform the synthesis of strictly size-controlled and monodisperse ZnO NPs in the presence of stabilizer due to poor solubility and high concentration of intermediate Zn(OR)_2_ formed in reaction system. This reaction system completely differs from that previously reported by Chaudret and co-workers for the synthesis of size- and shape-controlled crystalline ZnO NPs at room-temperature [51,52].

To further corroborate alkyl alcohol effect, thermal gravimetric analysis of intermediates Zn(OR)_2_ (R = Me, Et, *i*Pr and *n*Bu) was carried out to monitor their thermal decomposition temperature (TDT). As viewed in Figure S2 (ESI), the starting TDT markedly depends upon alkoxyl group of Zn(OR)_2_. The ordering of decomposition from low to high temperature is Zn(O*i*Pr)_2_ < Zn(O*n*Bu)_2_ < Zn(OEt)_2_ < Zn(OMe)_2_ < Zn(OGc). High TDT of Zn(OGc) has been reported previously [78]. For complexes Zn(OMe)_2_ and Zn(OGc), high TDT should be attributed to the polymeric structure of Zn(OMe)_2_ and the quite stable chelated structure of Zn(OGc), respectively. This result is in agreement with the experimental phenomena observed. More important is that high TDT probably influences the rate of growth of ZnO NPs and thereby results in the formation of different particle sizes and various morphologies.

Moreover, in order to confirm the formation of intermediate Zn(OR)_2_ and their compositions before thermal decomposition, the separated intermediates were dried and characterized by elemental analysis and IR spectrum. The results show that IR spectra (Figure S1, ESI) and the composition of the corresponding intermediate are quite similar to that of correspondingly fresh prepared zinc alkoxide molecule. For example, intermediate Zn(OEt)_2_, the contents of C, H and N are 30.57, 6.59 and 0.07 wt %, respectively. It is in good agreement with fresh Zn(OEt)_2_ prepared by the reaction of Zn[N(SiMe_3_)_2_]_2_ with anhydrous ethyl alcohol in hexane. For the synthesis of ZnO_-OR_ (R = *i*Pr, *n*Bu and Gc), the contents of C, H and N of intermediate are also in good agreement with the corresponding organozinc molecular precursor Zn(OR)_2_ (R = Et, *i*Pr, *n*Bu) and Zn(OGc), demonstrating the formation of intermediate zinc alkoxide. Note that intermediate did not contain any stabilizer or organic solvent after washing with the corresponding alkyl alcohol several times and drying. 

**Figure 4:**
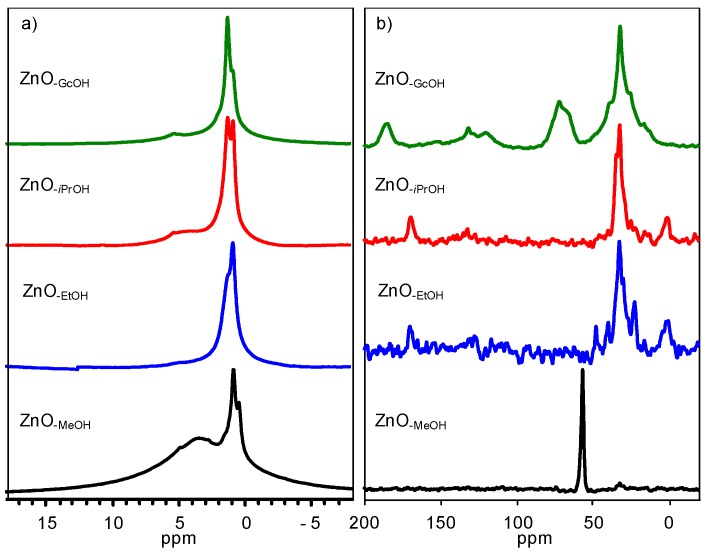
Solid-state (**a**) ^1^H and (**b**) ^13^C NMR spectra of as-made ZnO NPs obtained by using different alkyl alcohols as reactants under an identical condition except for ZnO_-GcOH_.

Furthermore, the chemical composition of as-made ZnO NPs was determined using the solid-state ^1^H and ^13^C NMR spectra, elemental analysis and infrared resonance spectra. All crystalline ZnO_-ROH_ NPs still contain high carbon contents ranging from 6 to 30 wt % (not shown). Due to low resolution of solid-state ^1^H NMR spectra, only chemical shifts of hydrogen atoms at δ = 0.6–5.5 ppm attributed to –CH_3_, –CH_2_, –CH=CH– groups from unreacted zinc precursor and stabilizer were observed for as-made ZnO_-ROH_ (R = Me, Et, *i*Pr and Gc) NPs (Figure 4a). In addition, for ZnO_-GcOH_ NPs, ^1^H NMR spectrum showed a very weak signal at δ = 11 ppm, confirming the presence of –COOH group from stabilizer oleic acid. However, high-resolution ^13^C spectra are markedly different (Figure 4b). For material ZnO_-MeOH_, solid-state ^13^C NMR spectrum clearly showed a single characteristic and strong signal at 56.3 ppm attributed to the undecomposed Zn(OMe)_2_ precursor but no characteristic peak of carbon from stabilizer oleylamine was observed. For crystalline ZnO_-EtOH_ and ZnO_-*i*PrOH_ NPs, the ^13^C NMR spectra indicate a series of signals at δ = 169.8 (C=O), 127.6 (CH=CH), 47.6–15.5 (CH_2_, CH_3_) and 0.7 ppm (trapped HN(SiMe_3_)_2_), revealing the presence of oleylamine stabilizer and by-products containing carbonyl group and the trapped by-product HN(SiMe_3_)_2_ and the absence of undecomposed zinc alkoxides. The formation of carbonyl group is relevant to intermediate of thermal decomposition of Zn(OR)_2_ (R = Et, *i*Pr) [54,80]. Furthermore, the appearance of C–H, C=C and C=O vibrations in IR spectra also further demonstrate the existence of stabilizer and incompletely decomposed by-product (such as Zn-carbonyl intermediate) from zinc precursor (Figure S3, ESI). Similarly, for ZnO_-GcOH_ material, the carbon signals at δ = 65.7 and 72.1 ppm are attributed to the incompletely decomposed zinc complex Zn[OCH_2_CH(OH)CH_2_O]. The characteristic carbon signals at δ = 184.8 (–CO_2_H), 139 and 118 (CH=CH_2_) and 130 (CH=CH) as well as δ = 14–35 ppm (CH_3_, CH_2_) belong to oleic acid and 1-octadecene.

**Figure 5:**
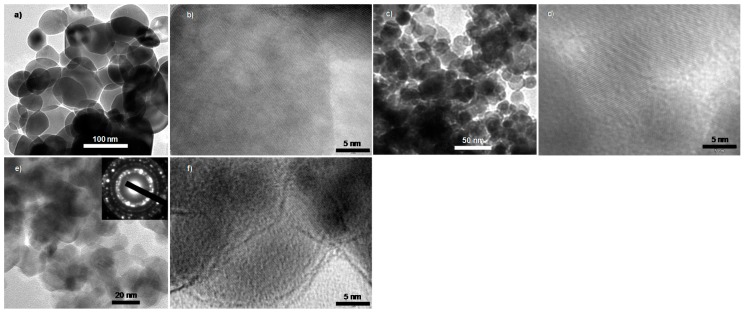
TEM images of organic composite-removed crystalline (**a**,**b**) ZnO_-MeOH_, (**c**,**d**) ZnO_-EtOH_ and (**e**,**f**) ZnO_-*i*PrOH_. The inset in (**e**) is corresponding SAED pattern.

Unsurprisingly, all organic composites in as-made ZnO_-ROH_ NPs can be completely removed by calcinations at 500 °C for 4 h. Representative TEM images for the calcined ZnO_-ROH_ (R = Me, Et and *i*Pr) are shown in Figure 5. A typically spherical morphology was preserved and an average particle size is 57.7 ± 1.5 nm for calcined ZnO_-MeOH_, 17.6 ± 0.6 nm for calcined ZnO_-EtOH_ and 12.3 ± 0.4 nm for calcined ZnO_-*i*PrOH_, respectively. Note that particle sizes markedly increased after high-temperature calcinations due to aggregation and growth of small particles. In addition, high crystalline structures with grain boundaries can be observed anywhere (Figure 5b,d,f). The selected area electron diffraction pattern of calcined ZnO_-*i*PrOH_ NPs reveals a typical würtzite-structured ZnO with *P6*_3_*mc* symmetry (Figure 5e, inset). Furthermore, highly well-resolved diffraction peaks in Figure S4 (ESI) also confirmed that hexagonal structure of crystalline ZnO_-ROH_ (R= Me, Et, *i*Pr, *n*Bu) were preserved after high-temperature calcinations but the intensity of diffraction peaks were much higher than the parent materials, implying that increasing crystalline particle size enhanced the intensity of diffraction peaks. The crystalline particle size calculated by Scherrer formula is 55.3 nm from the (100) plane for the spherical ZnO_-MeOH_, 19.1 nm from the (100) plane for the spherical ZnO_-EtOH_, 14.6 nm from the (100) plane for sphere-like ZnO_-*i*PrOH_ and 16.3 nm from the (100) plane for ZnO_-*n*BuOH_. These results are in good agreement with data measured by TEM images.

#### 2.1.3. Zinc Precursor Effect

**Figure 6:**
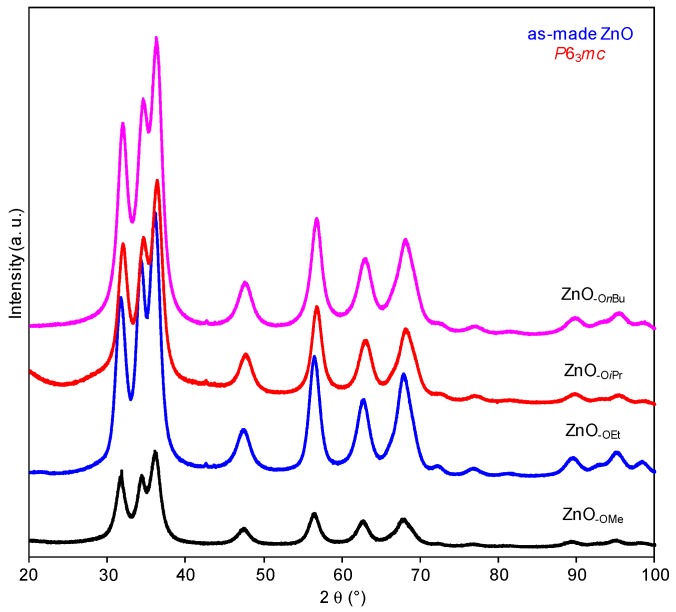
Wide-angle PXRD patterns of as-made ZnO_-OR_ NPs derived from directly thermal decomposition of fresh-prepared Zn(OR)_2_ (R = Me, Et, *i*Pr and *n*Bu) in the presence of oleylamine at 240 °C for 2 h.

As above-mentioned, the thermal decomposition of in-situ formed intermediates Zn(OR)_2_ (R = Me, Et, *i*Pr and *n*Bu) derived from the reaction of alkyl alcohol with Zn[N(SiMe_3_)_2_]_2_ markedly affect size and morphology of as-made ZnO_-ROH_ NPs in the presence of oleylamine. In order to corroborate this hypothesis, the directly thermal decomposition of fresh-prepared zinc precursors Zn(OR)_2_ (R = Me, Et, *i*Pr and *n*Bu) was performed in the presence of oleylamine to fabricate corresponding ZnO_-OR_ (R = Me, Et, *i*Pr and *n*Bu) NPs. The high crystalline würtzite-structure ZnO with *P6*_3_*mc* symmetry was corroborated by PXRD patterns (Figure 6), indicative of same structure as ZnO_-ROH_ NPs prepared by using thermal decomposition of in-situ formed zinc alkoxide. But TEM analyses confirm that particle size and morphology of ZnO_-OR_ NPs are quite different compared to corresponding ZnO_-ROH_ materials.

**Figure 7:**
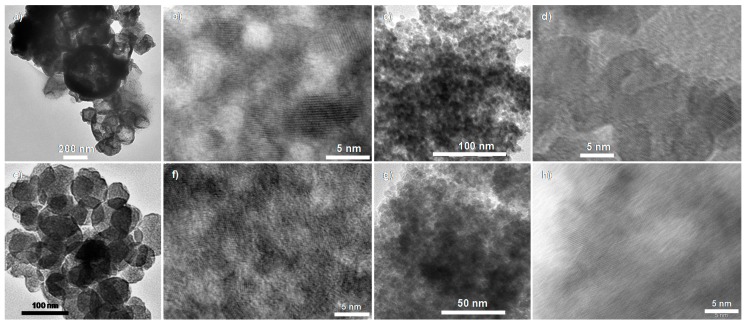
TEM images of ZnO_-OR_ NPs, for (**a**,**b**) ZnO_-OMe_, (**c**,**d**) ZnO_-OEt_, (**e**,**f**) ZnO_-O*i*Pr_ and (**g**,**h**) ZnO_-O*n*Bu_.

**Figure 8:**
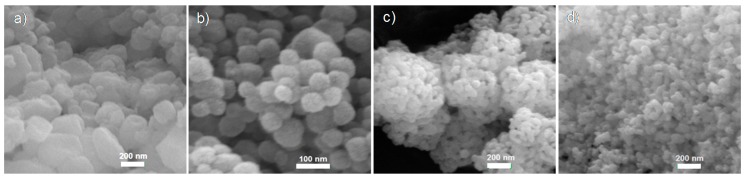
The representative SEM images of obtained ZnO NPs by the directly thermal decomposition and calcinations of Zn(OMe)_2_ and Zn(O*i*Pr)_2_; (**a**) ZnO_-OMe_; (**b**) ZnO_-O*i*Pr_; (**c**) ZnO_-Me_; and (**d**) ZnO_-*i*Pr_.

As viewed in Figure 7, all ZnO_-OR_ NPs with sphere-like or cuboid-like morphology show different sizes. Obviously, ZnO_-OMe_ particles are composed of hollow spheres with average diameter of 113.8 ± 12.2 nm and cuboid-like particles with different sizes in length and width (Figure 7a) to compare with that of ZnO_-MeOH_ (Figure 2). In fact, hollow spheres consist of very small particles (Table 1, the calculated particle size is 5.3 nm from the (100) reflection of XRD pattern). A representatively characteristic TEM image of ZnO_-OMe_ nanocrystals is shown in Figure 7b, revealing a typical crystalline structure. As viewed in Table 1, for crystalline ZnO_-OEt_ nanocrystals, the average particle size measured by TEM image is 5.4 ± 0.1 nm (Figure 7c,d). This result is close to 4.8 nm calculated by the Scherrer formula. For ZnO_-O*i*Pr_, the average size of spherical aggregation is 56.2 ± 1.8 nm (Figure 7e). These aggregations consist of crystalline nanoparticle with an average size of 4.2 ± 0.6 nm (Figure 7f, particle size calculated by the Scherrer formula is 4.8 nm from the (100) plane). For ZnO_-O*n*Bu_ NPs, the average particle size is 4.5 ± 0.2 nm and the lattice fringes can be observed (Figure 7g,h). Moreover, for ZnO_-OMe_ and ZnO_-O*i*Pr_ NPs, the shapes observed by SEM images (Figure 8a,b) are in accordance with that of TEM images observed under a low magnification.

On the basis of above-mentioned characterizations, we found that use of two different approaches based on thermal decomposition of in-situ-synthesized zinc alkoxides and directly thermal decomposition of fresh-prepared zinc alkoxides under an identical condition led to different results for preparing ZnO nanocrystals. With increasing carbon chain, the results trend to identical, such as ZnO_-*n*BuOH_ and ZnO_-O*n*Bu_. For ZnO_-ROH_ (R = Et, *i*Pr, *n*Bu) series, the calculated and measured average particle sizes are uniform and gradually decrease with increasing carbon atoms of alkoxyl group but ZnO_-MeOH_ makes an exception. For ZnO_-OR_ (R = Me, Et, *i*Pr, *n*Bu) series, no remarkable tendency is observed on the change of particle size and shape. For ZnO_-MeOH_ and ZnO_-OMe_, and ZnO_-*i*PrOH_ and ZnO_-O*i*Pr_, two approaches indicate a completely opposite phenomenon on the size of spherical aggregations. These differences can be contributed to two causes, one is that the in-situ formed zinc alkoxide can better disperse in oleylamine solvent except for {Zn(OMe)_2_}*_n_* and the fresh-prepared zinc alkoxides cannot better disperse in oleylamine solvent. Second is the change of different TDT-induced growth rate of zinc oxide from zinc alkoxides. Synergism of two aspects affects particle sizes and morphologies of as-made ZnO NPs. For example, Zn(OMe)_2_ showed a complicatedly thermal decomposition steps that thereby induced the formation of particles with different growth rate and aggregated together with different shapes and poor dispersion in oleylamine also enabled them to readily form aggregations.

**Figure 9:**
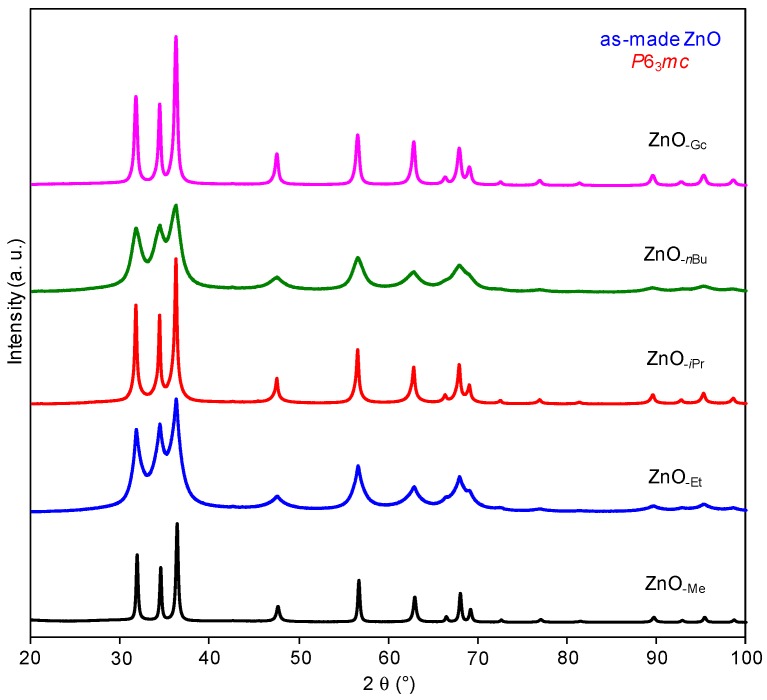
PXRD patterns of ZnO_-R_ NPs obtained by the direct calcinations of precursors Zn(OR)_2_ (R = Me, Et, *i*Pr, *n*Bu and Gc) at 500 °C for 4 h.

As a comparison, the direct calcination of Zn(OR)_2_ (R = Me, Et, *i*Pr, *n*Bu and Gc) at 500 °C for 4 h generated ZnO_-R_ NPs showed crystalline ZnO structures with 3D hexagonal symmetry, which were confirmed by PXRD analysis (Figure 9) and TEM images (Figure S5, ESI). The results reveal that the high-temperature calcination is obviously beneficial to the formation of crystalline ZnO NPs with the large particle size. According to Scherrer formula, the calculated particle size is 34.1 nm for ZnO_-Me_, 7.5 nm for ZnO_-Et_, 28.4 nm for ZnO_-*i*Pr_, 5.4 nm for ZnO_-*n*Bu_ and 21.3 nm for ZnO_-Gc_ from the (100) plane of XRD patterns (Table 1). Moreover, as a representative comparison, SEM images (Figure 8) between ZnO_-OMe_ and ZnO_-Me_ and ZnO_-O*i*Pr_ and ZnO_-*i*Pr_ displayed various morphologies for ZnO_-R_ series. As viewed in Figure 8, large particles of ZnO_-Me_ are composed of unnumbered small spherical particles. This result is quite similar to that of as-made ZnO_-MeOH_ NPs, indirect indicating poor dispersibility of precursor [Zn(OMe)_2_]*_n_* in solvents. Hence, three different approaches in the present study were used to prepare ZnO NPs, clearly revealing that their morphologies, crystalline particle sizes and the aggregation behaviour strongly depend on the zinc precursors and synthetic methods. These investigations are in agreement with the results reported previously [37]. Thermal decomposition and CVD method often lead to the formation of aggregation of ZnO with various particle sizes and with or without regular shapes from different organozinc precursors [34,54,59,60,81].

### 2.2. Electron Paramagnetic Resonance (EPR) Spectra of a Series of Nano-Structured Zinc Oxide Particles ZnO_-ROH_, ZnO_-OR_ and ZnO_-R_ (R = Me, Et, iPr, nBu, Gc)

Different synthesis methods and various zinc precursors often induce the change of physical and chemical properties of obtained nanostructured materials due to different defects. In general, for ZnO NPs, the types of defect centres include zinc vacancies (V_Zn_, V_Zn_^+^, V_Zn_^2+^), zinc on interstitial sites (Zn_i_, Zn_i_^+^, Zn_i_^2+^), oxygen vacancies (V_O_, V_O_^−^, V_O_^2−^, V_O_^+^, V_O_^2+^) and oxygen on interstitial sites (O_i_, O_i_^−^, O_i_^2−^). In these intrinsic defect centres only V_Zn_, V_Zn_^+^, Zn_i_^+^, O_i_, O_i_^−^, V_O_, V_O_^−^, V_O_^2−^ and V_O_^+^ defect centres can be monitored by EPR spectra [82], especially, oxygen vacancies [80,81,83,84,85,86,87,88]. Hence, the EPR investigation of pure ZnO materials are not surprising. Herein, as a standard field marker, polycrystalline DPPH with electron spin *g*-factor (*g* = 2.0036) was used for the exact determination of the magnetic field offset. For ZnO materials, the previous reports have discussed in detail on that different defects generated the appearance of different signals at *g* = 1.96 and 2.00 in EPR spectra, although these assignments of the EPR signals have caused much controversy [80,81,89,90]. At present, the low-field signal at *g* = 2.00 is often assigned to an unpaired electron on an oxygen vacancy site [81,90,91] and high-field signal with *g* = 1.96 is either attributed to shallow donor centres such as ionized impurity atoms in the crystal lattices of ZnO [91,92,93,94,95,96,97,98], or to unpaired electrons on oxygen vacancies in some cases. When tetrameric methylzinc *tert*-butoxide [MeZnO*t*Bu]_4_ or [MeZnO*i*Pr]_4_ complex was used as a molecular precursor to prepare ZnO NPs, Driess and co-workers emphasized that EPR signals with *g* ~ 2.000 and 1.96 were attributed to oxygen vacancies with a single trapped electron (V_O_^+^) and impurity atoms in the ZnO lattices, respectively [80,81]. It is worth noting that with increasing calcination temperature EPR signal with *g* ~ 2.000 decreased until completely disappeared.

**Figure 10:**
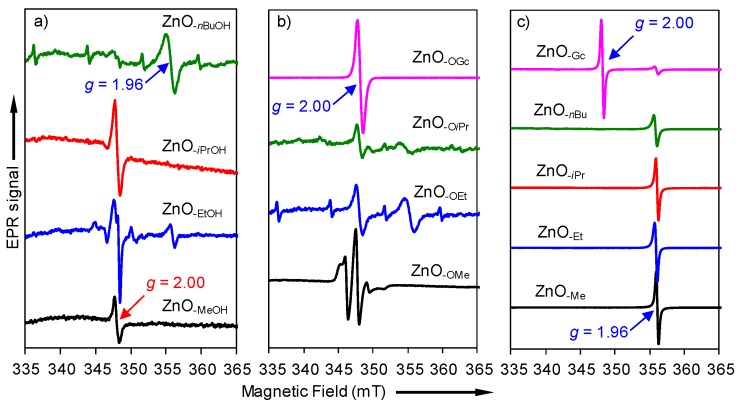
Room temperature EPR spectra of ZnO NPs derived from different synthetic approaches, for (**a**) ZnO_-ROH_; (**b**) ZnO_-OR_; and (**c**) ZnO_-R_ (R = Me, Et, *i*Pr, *n*Bu and Gc).

In the present study, the X-band EPR properties of ZnO NPs prepared by three different approaches are investigated and their spectra are shown in Figure 10. All electron spin *g*-factors of all ZnO particles are listed in Table 1. For ZnO_-ROH_ series, EPR spectra of ZnO_-MeOH_ and ZnO_-*i*PrOH_ NPs only show a signal with an electron spin *g*-factor around 2.00, suggesting an unpaired electron trapped on an oxygen vacancy site [80,81,90]. However, a series of weak or strong signals at *g* = 2.02, 2.00, 1.99 and 1.96 for ZnO_-EtOH_ and at *g* = 2.12, 2.07, 2.03, 2.00, 1.98, 1.96 and 1.94 for ZnO_-*n*BuOH_ NPs, are identified, respectively, revealing the co-existence of unpaired electrons on oxygen vacancies and impurity atoms in the ZnO lattices (Figure 10a). This phenomenon has been reported by Driess and co-workers due to oxygen vacancies and the type and concentration of impurity heteroatoms trapped in the ZnO lattices during the growth of ZnO particles [81]. In fact, the above-mentioned ^13^C NMR spectra have clearly confirmed doping of impurity heteroatoms in ZnO in our ZnO NPs. Additionally, note that when the fresh-prepared Zn(OR)_3_ (R = Me, Et and *i*Pr) was used as the zinc precursor and other conditions are identical for the fabrication of ZnO_-OR_ NPs, the X-band EPR spectra clearly verified the formation of different defect centres with *g*-factors of 2.01, 2.00, 1.99 for ZnO_-OMe_, of the ranging from 2.12 to 1.90 for ZnO_-OR_ (R = Et, *i*Pr), respectively. Weak or strong EPR signals revealed the ratio of predominant and secondary defect centres (Figure 10b) and their relevance to use of organozinc molecular precursor, for example, starting precursor or intermediate is Zn(OMe)_2_, no EPR signal with *g* ~ 1.96 appeared, if with Zn(OEt)_2_ two signals with *g* ~ 2.00 and 1.96 appeared in EPR spectra.

As a comparison, a series of ZnO_-R_ materials prepared by the direct calcination of Zn(OR)_3_ (R = Me, Et, *i*Pr and *n*Bu) at 500 °C for 4 h only exhibited one signal with *g*-factor of 1.96 in X-band EPR spectra (Figure 10c), except ZnO_-GC_ that indicated two signals with *g*-factor of 2.00 and 1.96. The previous researches have carefully investigated the case of the appearance of the EPR signals with *g* = 2.00 and 1.96 in ZnO nanocrystals. The former is attributed to surface defects (a singly ionized Zn vacancy or an unpaired electron trapped in an oxygen vacancy site or stable O^2−^ vacancy) due to the formation of core-shell structured ZnO nanocrystals during the milling process [82,88] but this attribution is still controversial and the latter is assigned to a singly ionized oxygen vacancy (V_O_^+^) which has been confirmed by UV-light irradiation that produced an enhanced EPR signal at *g* = 1.96 [99] or is relevant to oxygen vacancy concentration or impurity atoms effect [81,86]. In some cases, low-field paramagnetic signal (*g* = 2.00) in ZnO materials can also be attributed to oxygen vacancies with a single trapped electron (V_O_^+^) [81]. Moreover, ZnO materials maybe have interstitial zinc, V_O_^−^, V_Zn_ and O_i_ defects but V_Zn_ and O_i_-type defect are thermodynamically stable in the ZnO crystal lattices at higher oxygen partial pressure. In our ZnO NPs, no clear core-shell structure is observed and no special controlled condition except argon environment was performed during the preparation of ZnO NPs, hence we suggest that EPR signal at *g* = 1.96 is related to oxygen vacancy and impurity atoms (C, Si or N) in the ZnO lattices. Note that the calcinations of ZnO_-R_ (R = Me, Et, *i*Pr, *n*Bu) at 500 °C for 4 h formed ZnO particles did not show EPR signal at *g* = 2.00. This result is in better agreement with that the signal at *g* = 2.00 appears only at lower temperatures [81]. But the EPR spectra of ZnO_-GC_ NPs indicates two signals at *g* = 2.00 and 1.96, clearly confirming that the types of defects are probably influenced by the coordination modes and the structure of coordination ligand of zinc precursor during calcinations. On the basis of above-mentioned discussions, the formation of defect centres or defect chemistry in ZnO NPs depends not only on the use of the original alkyl alcohols but also on the synthesis conditions [37,100,101]. Physical-chemical property of materials reflects the alteration of structure of materials in composition and defect chemistry.

### 2.3. Optical Properties of a Series of Nano-Structured Zinc Oxide Particles ZnO_-ROH_, ZnO_-OR_ and ZnO_-R_ (R = Me, Et, iPr, nBu, Gc)

#### 2.3.1. UV-Vis Spectra

**Figure 11:**
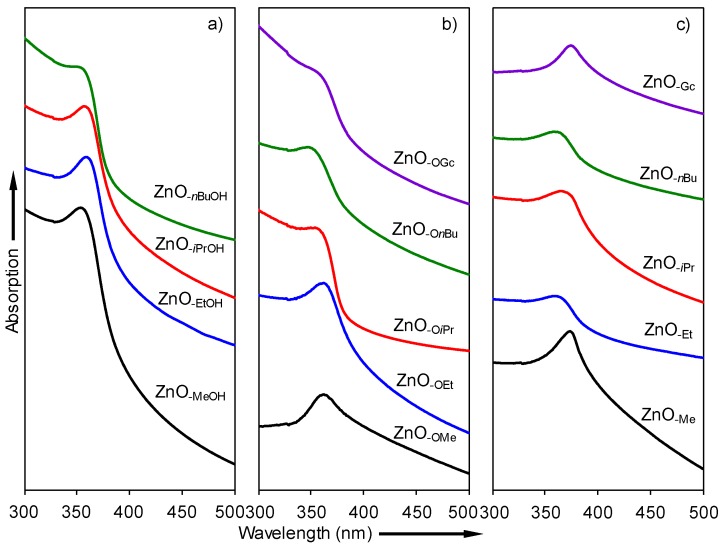
Size-dependent UV-Vis absorption spectra of (**a**) ZnO_-ROH_; (**b**) ZnO_-OR_; and (**c**) ZnO_-R_ (R = Me, Et, *i*Pr, *n*Bu and Gc) NPs.

For a series of ZnO NPs, size-dependent ultraviolet-visible (UV-Vis) absorption spectra are shown in Figure 11. The band centred at 352, 358, 356 and 351 nm for nanostructured ZnO_-MeOH_, ZnO_-EtOH_, ZnO_-*i*PrOH_ and ZnO_-*n*BuOH_ were respectively observed, indicating the occurrence of blue shift in the ZnO_-ROH_ NPs (Figure 11a) compared to bulk ZnO (380 nm) due to size quantization effect caused by the confinement of the movement of electrons [102]. As listed in Table 1, position of band from UV-Vis absorption spectra indirectly reflects particle sizes of ZnO NPs. The results are in agreement with particle sizes measured by TEM microscopy and calculated by Scherrer formula (ZnO_-EtOH_ > ZnO_-*i*PrOH_ > ZnO_-MeOH_ > ZnO_-*n*BuOH_). Similar blue shift phenomena were also observed for ZnO_-OR_ and ZnO_-R_ (R = Me, Et, *i*Pr, *n*Bu and Gc) (Figure 11b,c) but UV-Vis adsorption spectra of ZnO_-Me_, ZnO_-*i*Pr_ and ZnO_-Gc_ are very similar to that of bulk ZnO due to large particles under high temperature condition. It is worth noting that the occurrence of blue shift depends on not only synthesis approaches but also nature and structure of precursors in the present reaction system.

#### 2.3.2. Photoluminescence (PL) Property

**Figure 12:**
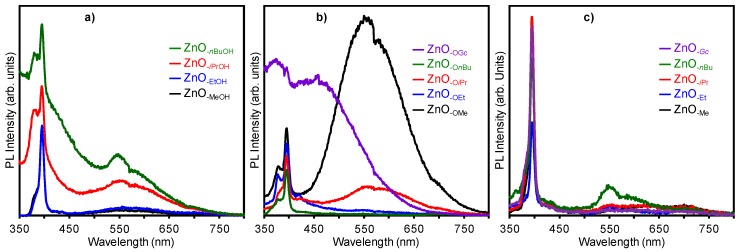
Photoluminescence spectra of (**a**) ZnO_-ROH_; (**b**) ZnO_-OR_; and (**c**) ZnO_-R_ (R = Me, Et, *i*Pr, *n*Bu and Gc) NPs derived from different synthetic approaches.

**Figure 13:**
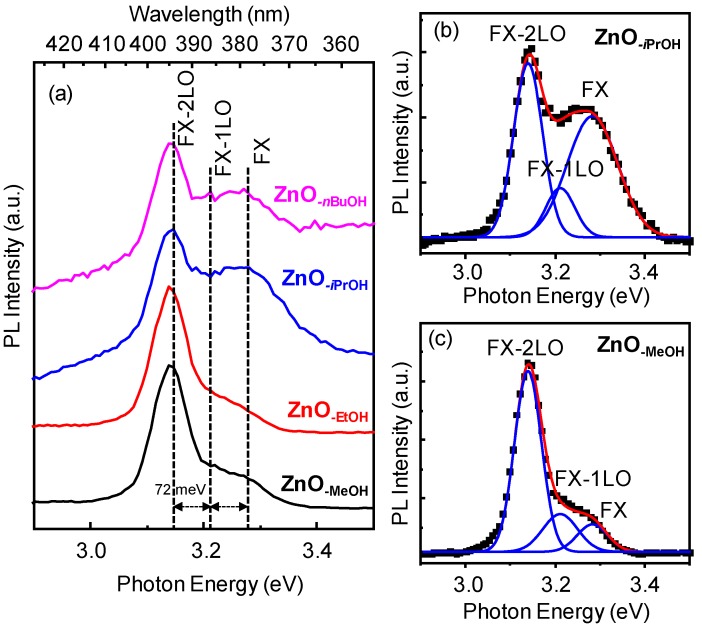
(**a**) PL spectra of ZnO_-ROH_ (R = Me, Et, *i*Pr and *n*Bu) NPs in the UV range; (**b**,**c**) spectral fitting of the peaks for ZnO_-*i*PrOH_ and ZnO_-MeOH_, respectively.

To further characterize the optical natures of a series of ZnO-ROH, ZnO-OR and ZnO-R (R = Me, Et, *i*Pr, *n*Bu and Gc) NPs, photoluminescent spectra were recorded (Figure 12). All ZnO NPs clearly show a strong peak nearly at 394 nm (3.14 eV) with excitation wavelength of 290 nm. Note that owing to use of different organozinc precursors, PL spectra of ZnO_-ROH_ (Figure 12a) and ZnO_-OR_ (R = Me, Et, *i*Pr and *n*Bu) NPs (Figure 12b) also show a shoulder peak at about 378 nm (3.28 eV) and the intensity of shoulder peak almost keeps same for the ZnO_-ROH_ (R = Me, Et) NPs, generally increases for the ZnO_-ROH_ (R = *i*Pr, *n*Bu) and ZnO_-OR_ (R = Me, Et, *i*Pr and *n*Bu) NPs. After carefully checked our experimental data and compared with those previously reported spectra [103,104,105,106], we concluded that the PL peaks in the UV range from 370 to 400 nm are due to near band gap emissions and can be considered as exciton in origin, although it is slightly less than 3.37 eV at room temperature [1,2]. In general, the band gap emission at room temperature is dominated by phonon replica of free exciton due to strong exciton-phonon coupling [1,2,67]. This phenomenon was also observed in our experiments. As an example, the spectral analyses of a series of ZnO_-ROH_ (R = Me, Et, *i*Pr and *n*Bu) NPs are shown in Figure 13. After fitting the PL spectra in the range from 2.9 to 3.5 eV, we find three peaks located at 3.139 eV (394 nm), 3.211 eV (386 nm) and 3.283 eV (378 nm). The energy difference of these peaks is about 72 meV, which corresponds to the longitudinal optical (LO) phonon energy in ZnO. Therefore, we can assign the peak at 3.283, 3.211 and 3.139 eV as the free exciton (FX) emission, the first order phono-assisted emission (free exciton-1 LO, FX-1LO) and the second order phono-assisted emission (free exciton-2 LO, FX-2LO), respectively. As shown in Figure 13b,c, for different ZnO NPs, the intensity ratio of these contributions is different.

Indeed, the observed PL peaks show less photon energy compared to the bandgap of the bulk ZnO. The PL of ZnO nanostructures previously reported at room temperature showed that near band gap emission was much less energy than the band gap (3.37 eV) of bulk ZnO. For instance, Wang and co-workers early reported that the ultrathin ZnO nanobelts (6 nm) exhibited a near band edge emission at 373 nm (3.32 eV) [107], while the normal ZnO nanobelts showed a peak at 387 nm (3.2 eV). In addition, one review paper also elucidated the PL of ZnO nanostructures varying from 372 to 390 with different shapes [2]. These below the bulk band gap (3.37 eV) of ZnO are probably caused by different morphologies [65], surface defects and impurity [108], as well as size effect [109], which can affect the near band edge emission. Hence the red shift of the near band edge emission of ZnO NPs in the present study can be attributed to the influences of these factors, such as ZnO_-ROH_, ZnO_-OR_ and ZnO_-R_ series prepared by using zinc precursors with increasing carbon chain length of alkoxyl groups, possessing different defects caused by the preparation process. These results are probably related to that the presence of stabilizer and the incomplete decomposition of zinc alkoxides led to the doping of impurity into the ZnO lattices and surface defects (Figure 4). Based on the above-mentioned analysis of the alteration of the origin of the UV peak in these nanocrystalline ZnO particles, the change of near band gap emission can be readily understood by free excitonic or defect-related emission, surface impurity and defects depending on the synthesis method [5,64,65,66]. When chelated zinc glycerate was used as the zinc precursor, the obtained ZnO_-OGc_ NPs showed a strong photoluminscent peak at 378 nm and a broad peak centred at 450 nm (Figure 12b) and the peak at 450 nm is unclear. However, besides the fundamental ZnO emission, some weak or strong peaks with subgap energies at 550 nm (2.25 eV, “green” luminescence) [110] and 600 nm (2.06 eV) are detected in the photoluminescent spectra (Figure 12), especially, for ZnO_-ROH_ and ZnO_-OR_ (R = Me, *i*Pr) and ZnO_-R_ (R = *i*Pr, *n*Bu) NPs. The luminescent peaks can be attributed to oxygen vacancies at 550 nm and surface interstitial oxygen at about 600 nm [87]. This result is in good agreement with the energy level of oxygen vacancies calculated by Fu and co-workers [111]. Based on above-mentioned analysis, the intensity of luminescent peaks of ZnO NPs mainly depends on the ratio or concentration of predominant and secondary defect centres, such as zinc on interstitial sites (Zn_i_), oxygen vacancies, oxygen on interstitial sites (O_i_) and impurity heteroatoms. In fact, the formation of these defect centres are often affected by zinc precursor used, the presence of stabilizer and synthesis conditions [81,86,100,101].

Based on above-mentioned EPR investigations and PL spectra, we try to explain the relationship between EPR signals (*g* = 2.00, 1.96) and photoluminescence spectra of ZnO (λ = 378, 550, 600 nm) by having obtained luminescence property. In general, the types of defects or vacancies in a material often influence the emission spectrum of material. However, for the present ZnO NPs, we find that it is difficult to establish a direct relation between EPR signals and luminescence behaviour, for example, ZnO_-MeOH_ NPs showed a strong EPR signal at *g* = 2.00 which designed to an unpaired electron trapped on an oxygen vacancy site but PL spectrum did not show any strong photo-luminescent peak at λ = 550 nm as “green” luminescence, ZnO_-EtOH_ NPs showed two EPR signals at *g* = 2.00 and 1.96 but its luminescence behaviour is quite similar to that of ZnO_-MeOH_. Note that ZnO_-*i*PrOH_ and ZnO_-*n*BuOH_ NPs showed a completely different surface defect type (oxygen vacancy (*g* = 2.00) and doping of impurity or surface interstitial oxygen (*g* = 1.96), Figure 10) but they showed a quite similar photoluminescence property reflected by “green” luminescence peak at λ = 550 nm and impurity-doping or surface interstitial oxygen-induced luminescence peak at λ = 600 nm from the PL spectrum (Figure 12). For ZnO_-OR_ and ZnO_-R_ series, a similar situation are also observed (Figure 12). These results confirm that for our ZnO NPs no unified regulation can be found between EPR signals and photoluminescence behaviours. Hence, we think that these results clearly reveal the complication of defect chemistry and photoluminescence properties. In fact, our study is in good accordance with results previously reported [81,86,88].

## 3. Materials and Methods

### 3.1. General Consideration

For synthesis of organozinc compounds, all operations were performed with rigorous exclusion of air and moisture, using glovebox techniques (MB Braun MB150B-G-II; <1 ppm O_2_, <1 ppm H_2_O, argon) or the standard Schlenk technique. Anhydrous methanol (99.8%), anhydrous isopropanol (99.5%) and oleylamine (tech. 70%) were purchased from Sigma-Aldrich (St. Louis, MO, USA) and used as received. Absolute ethanol (99.5%, Sigma-Aldrich, St. Louis, MO, USA) and anhydrous *n*-butanol (99.8%, Sigma-Aldrich, St. Louis, MO, USA) were used after drying with 5 Å molecular sieves and distillation. Glycerol (ACS reagent, ≥99.5%, Sigma-Aldrich, St. Louis, MO, USA) was used after drying with 5 Å molecular sieves. Hexane solvent was purified by using Grubbs columns (MBraun SPS, solvent purification system) before using. Zn[N(SiMe_3_)_2_]_2_ were synthesized according to previously reported method with slight modification [75]. Zn(OR)_2_ (R = Me, Et, *i*Pr and *n*Bu) was prepared by the reaction of one mole of Zn[N(SiMe_3_)_2_]_2_ and two moles of alkyl alcohol in hexane [74].

### 3.2. Synthesis of Crystalline Zinc Oxide NPs

#### 3.2.1. The Preparation of ZnO_-ROH_ (R = Me, Et, *i*Pr, *n*Bu, Gc) Nanoparticles

ZnO nanoparticles were synthesized by thermal decomposition method. The typical preparation procedure is presented as follows. Synthesis of spherical crystalline ZnO_-MeOH_ nanoparticles: Oleylamine (4 mL) was added into a two-neck flask and degassed at 100 °C for 30 min, followed by the filling of argon. 4 mL of anhydrous methanol was then added under argon protection. After being stirred for 10 min, the solution of Zn[N(SiMe_3_)_2_]_2_ (3.00 g) in 12 mL of hexane was added dropwise by syringe rejection under vigorous stirring. White flocculent turbidity formed slowly. The following the mixture was refluxed at 100 °C for 1.5 h and then naturally cooled down to room temperature. The volatile matters were removed under vacuum and the final suspension was heated at a rate of 5 °C per minute to 240 °C and kept this temperature for 2 h. The resulting suspension was cooled down to room temperature, then 10 mL of methanol was added and stirred for several minutes. White precipitate was collected by centrifugation at a rate of 6000 rotations per minute for 10 min and washed twice with 5 mL of hexane. The precipitate was then dispersed in hexane. Part of separated precipitates was dried at 100 °C and used for analysis, denoted as ZnO_-MeOH_. 

Similarly, for materials ZnO_-EtOH_, ZnO_-*i*PrOH_ and ZnO_-*n*BuOH_, the detailed synthesis procedures are completely similar to that of ZnO_-MeOH_, except that different alkyl alcohol was used as a reaction solvent. But for the preparation of ZnO_-GcOH_ (GcOH = glycyl alcohol), the mixture of 1-octadecene and oleic acid was used as a mixed solvent, the decomposition temperature was 320 °C and other conditions are identical. 

#### 3.2.2. The Preparation of ZnO_-OR_ (R = Me, Et, *i*Pr, *n*Bu, Gc) Nanoparticles

The fresh-prepared Zn(OR)_2_ (R = Me, Et, *i*Pr and *n*Bu) was respectively used as a zinc molecular precursor and oleylamine was used as a reaction solvent. Reaction temperature and time are same as the preparation of ZnO_-MeOH_ NPs. The obtained ZnO material was denoted as ZnO_-OR_. For the ZnO_-OGC_ preparation, the mixed oleic acid and 1-octadecene was used as a high boiling-point solvent and heating temperature was 320 °C for 2 h.

#### 3.2.3. The Preparation of ZnO_-R_ (R = Me, Et, *i*Pr, *n*Bu, Gc) Nanoparticles

As a comparison, a series of zinc oxide materials denoted as ZnO_-R_ (R = Me, Et, *i*Pr, *n*Bu and Gc) were obtained by the direct calcination of fresh-prepared Zn(OR)_2_ (R = Me, Et, *i*Pr, *n*Bu and Gc) at 500 °C for 4 h (Temperature-controlled programme: (i) from room temperature to 500 °C with a heating rate of 5 °C per minute; (ii) 500 °C, 4 h; (iii) naturally cool down to room temperature).

### 3.3. Characterization

Powder X-ray diffraction (PXRD) analysis of the as-synthesized and stabilizer-free ZnO nanostructured materials were recorded on a Bruker Advance D8 instrument in the step/scan mode using monochromatic CuK_α_ radiation (λ = 1.5406 Å). The wide-angle diffractograms were collected in the 2θ range of 10–100 °C with a scanspeed of 5. The crystallite size of the NPs is evaluated from X-ray powder diffraction data using Scherrer formula *D*_hkl_ = *K*λ/(βcosθ), where *D*_hkl_ is the mean size of the ordered crystalline grain size, K is a dimensionless shape factor (0.89), λ is the X-ray wavelength of the Cu target (0.15406 nm), β is the full width at half the maximum (FWHM) intensity (in radians), θ is the Bragg angle (in degrees). Transmission electron microscopy (TEM) images were obtained on a JEOL JEM2100 microscopy (Akishima, Tokyo, Japan) with an operating voltage of 160 kV. For TEM measurement, a drop of fine powdery material suspended in ethanol (99.9%) under the ultrasonic vibration was loaded onto a holey carbon film on a square 400 mesh copper 3.05 mm grid. Scanning electron microscopy (SEM) images were recorded on a JEOL JSM-5900LV microscope (JEOL Ltd., Tokyo, Japan) accompanying with EDX system operated at an accelerating voltage of 15 kV. All SEM images reported here are representative of the corresponding materials. Diffuse Reflectance Infrared Fourier-Transform (DRIFT) spectra of ZnO nanostructured materials were recorded on a Thermo Scientific Nicolet 6700 FTIR spectrometer (Waltham, MA, USA) with KBr reference (256 scans, a resolution of 4 cm^−1^) in the range of 4000–400 cm^−1^. ^1^H and ^13^C CP MAS NMR spectra were obtained at room temperature on a Bruker DSX 200 instrument (Billerica, MA, USA) equipped with magic angle spinning (MAS) hardware and using ZrO_2_ rotor with an inside diameter of 3 mm. Adamantine was used as a reference (^1^H: 1.76 and 1.87 ppm; ^13^C: 28.46 and 37.85 ppm). Ultraviolet-visible (UV-Vis) absorption spectra were taken by using a Perkin Elmer Lambda 35 spectrometer (Waltham, MA, USA). Photoluminescence (PL) studies were conducted using a Varian Cary Eclipse fluorescence spectrophotometer (Palo Alto, CA, USA) with a Xe lamp at room temperature with excitation wavelength of 290 nm. X-band (10 GHz) electron paramagnetic resonance (EPR) spectra were obtained on a Bruker ESR 300E spectrometer (Billerica, MA, USA) at ambient temperature. The magnetic field was determined using a nuclear magnetic resonance gaussmeter (Villebon-Sur-Yvette, France). As a standard field marker, polycrystalline DPPH with electron spin *g*-factor (*g* = 2.0036) was used for the exact determination of the magnetic field offset. Elemental analyses of C, H and N were performed on an Elementar Vario EL III (Langenselbold, Germany). Thermogravimetric analyses (TGA) were obtained on a Netzsch STA 449F3 instrument (Selb, Germany) equipped with a quartz crucible at a heating rate of 2 K min^−1^ under Ar/O_2_/Ar atmosphere.

## 4. Conclusions

Crystalline ZnO nanoparticles have been successfully synthesized by using three different approaches—thermal decomposition of in-situ formed zinc alkoxide from the reaction of Zn[N(SiMe_3_)_2_]_2_ and various alkyl alcohol, thermal decomposition of fresh-prepared Zn(OR)_2_ (R = Me, Et, *i*Pr, *n*Bu, Gc) in the presence of stabilizer and the direct calcination of Zn(OR)_2_ (R = Me, Et, *i*Pr, *n*Bu, Gc) at 500 °C. The alteration of morphology, crystalline particle sizes, aggregation behaviour and defect chemistry of obtained ZnO NPs depend on not only organozinc precursor but also on synthesis condition. The change of adsorption peaks in UV-Vis spectra demonstrated particle size effect. Various EPR and photoluminescent spectra confirmed defect behaviour of zinc or oxygen vacancies or impurity heteroatoms in crystalline ZnO NPs. These results demonstrated the relationship between structure and property and the complication of the formation of defect chemistry and photoluminescence behaviour in ZnO NPs.

## Figures and Tables

**Table 1:** Particle size determined by TEM and XRD data and absorption peak from UV spectra.

Sample	Particle Size/nm ^a^	Particle Size/nm ^b^	Electron *g*-Factor	Absorption Edge/nm
ZnO_-MeOH_	5.9 ± 0.3	4.55; 4.86	2.00	352
ZnO_-EtOH_	11.7 ± 0.6	10.7; 10.2	2.02, 2.00, 1.99, 1.96	358
ZnO_-*i*PrOH_	7.5 ± 0.2	9.0; 8.3	2.00	356
ZnO_-*n*BuOH_	-	4.3	2.12, 2.07, 2.00, 1.96	351
ZnO_-GcOH_	-	3.8	-	-
ZnO_-OMe_	different sizes	5.3	2.01, 2.00, 1.99	364
ZnO_-OEt_	5.4 ± 0.1	4.8	2.12~1.90	361
ZnO_-O*i*Pr_	4.2 ± 0.6	4.8	2.12~1.90	347
ZnO_-O*n*Bu_	4.5 ± 0.2	5.1	-	346
ZnO_-OGc_	-	-	2.00	348
ZnO_-Me_	-	34.1	1.96	373
ZnO_-Et_	-	7.5	1.96	358
ZnO_-*i*Pr_	-	28.4	1.96	365
ZnO_-*n*Bu_	-	5.4	1.96	358
ZnO_-Gc_	-	21.3	2.00, 1.96	373

Note: ^a^ Particle size was measured using TEM images; ^b^ Particle size was calculated using the Scherrer formula from (100) diffraction peak of XRD patterns.

## Supplementary Materials

The following are available online at http://www.mdpi.com/2079-4991/8/1/22/s1, Figure S1: DRIFT spectra of the separated and dried intermediate zinc alkoxides before thermal decomposition, Figure S2: Thermogravimetric analysis curve of zinc alkoxides, Zn(OR)_2_ (R = Me, Et, *i*Pr and *n*Bu), Figure S3: DRIFT spectra of as-made ZnO_-MeOH_, ZnO_-EtOH_, ZnO_-*i*PrOH_, ZnO_-BuOH_ and ZnO_-GCOH_ NPs (Left) and zinc alkoxides (Right), Figure S4: PXRD patterns of calcined ZnO_-ROH_ (R = Me, Et, *i*Pr and *n*Bu), Figure S5: High-resolution TEM images of the crystalline ZnO_-Me_ (Left) and ZnO_-*i*Pr_ (Right) NPs obtained by direct calcinations of Zn(OMe)_2_ and Zn(O*i*Pr)_2_.
