# Supplementary material for: Organozinc Precursor-Derived Crystalline ZnO Nanoparticles: Synthesis, Characterization and Their Spectroscopic Properties
Source: Nanomaterials (Basel). 2018 Jan 4;8(1):22. doi: 10.3390/nano8010022 (PMC5791109; doi:10.3390/nano8010022)
Supplement: Supplementary file 1 [file nanomaterials-08-00022-s001.pdf]

Supplementary Information

Organozinc Precursor-Derived Crystalline ZnO Nanoparticles: Synthesis, Characterization and Their Spectroscopic Properties

Yucang Liang [1,*], Susanne Wicker [1], Xiao Wang [2], Egil Severin Erichsen [3] and Feng Fu [4,*]

1 Institut für Anorganische Chemie, Eberhard Karls Universität Tübingen, Auf der Morgenstelle 18, 72076 Tübingen, Germany; susanne.wicker@ipc.uni-tuebingen.de
2 School of Physics and Electronics, Hunan University, Changsha 410082, China; xiao_wang@hnu.edu.cn
3 Laboratory for Electron Microscopy, University of Bergen, Allégaten 41, 5007 Bergen, Norway; Egil.Erichsen@mnfa.uib.no
4 College of Chemistry & Chemical Engineering, Yan'an University, Shaanxi Key Laboratory of Chemical Reaction Engineering, Yan'an 716000, China
* Correspondence: yucang.liang@uni-tuebingen.de (Y.L.); yadxfufeng@126.com (F.F.); Tel.: +49-07071-29-76216 (Y.L.); +86-911-2332037 (F.F.)

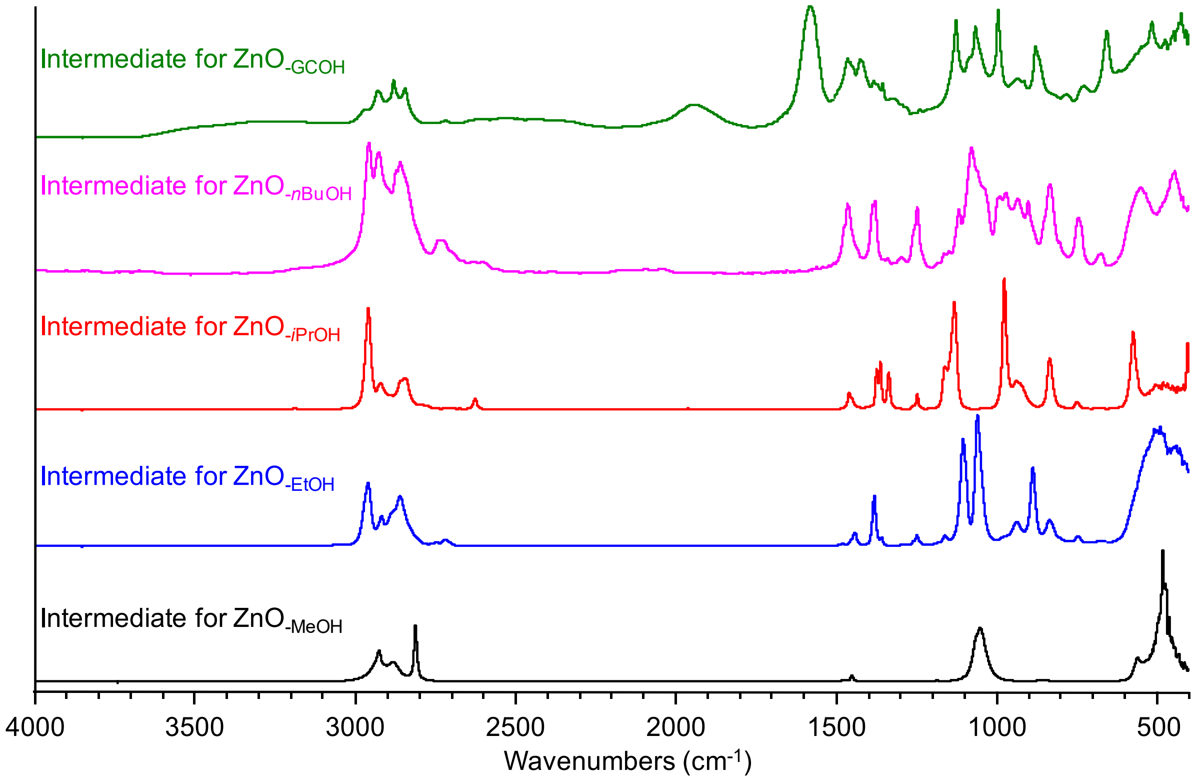

Figure S1. DRIFT spectra of the separated and dried intermediate zinc alkoxides before thermal decomposition.

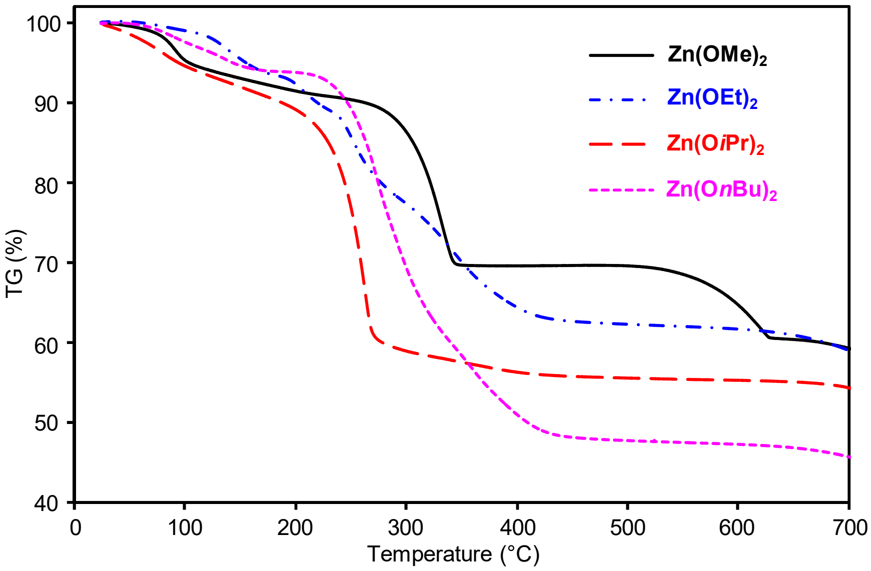

Figure S2. Thermogravimetric analysis curve of zinc alkoxides, $Zn(OR)_2$ (R = Me, Et, *i*Pr and *n*Bu).

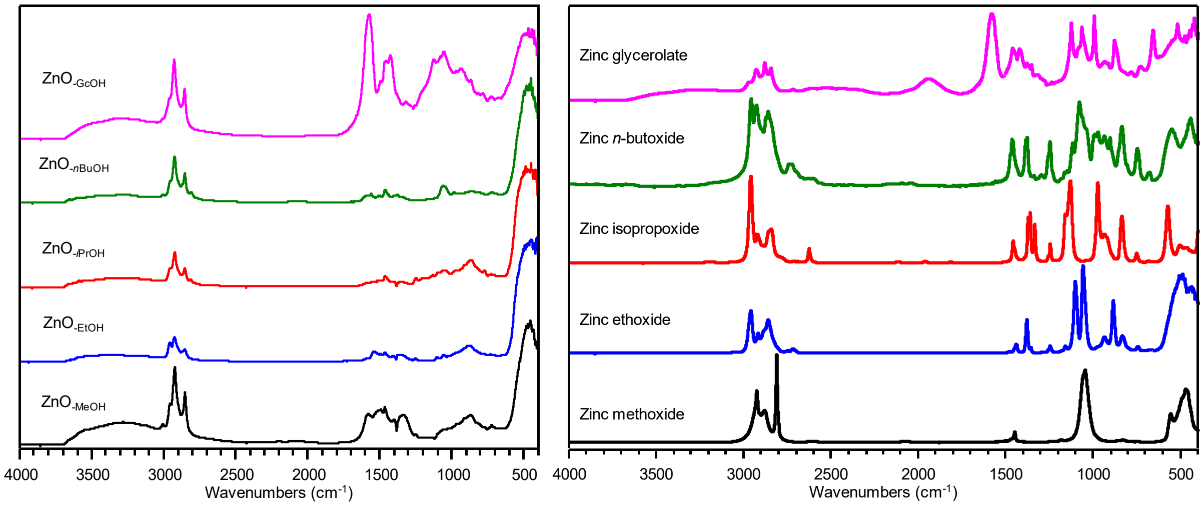

Figure S3. DRIFT spectra of as-made $ZnO_{\text{-MeOH}}$, $ZnO_{\text{-EtOH}}$, $ZnO_{\text{-}i\text{PrOH}}$, $ZnO_{\text{-}n\text{BuOH}}$ and $ZnO_{\text{-GcOH}}$ nanoparticles (Left), and zinc alkoxides (Right).

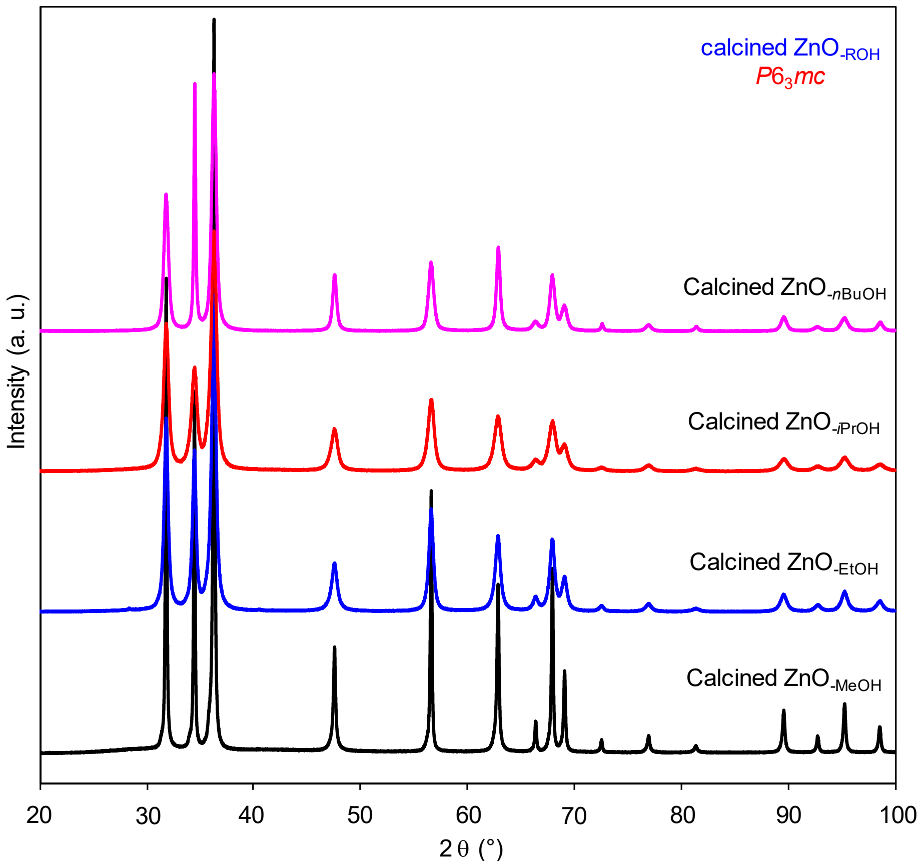

Figure S4. PXRD patterns of calcined ZnO_{-ROH} (R = Me, Et, *i*Pr and *n*Bu)

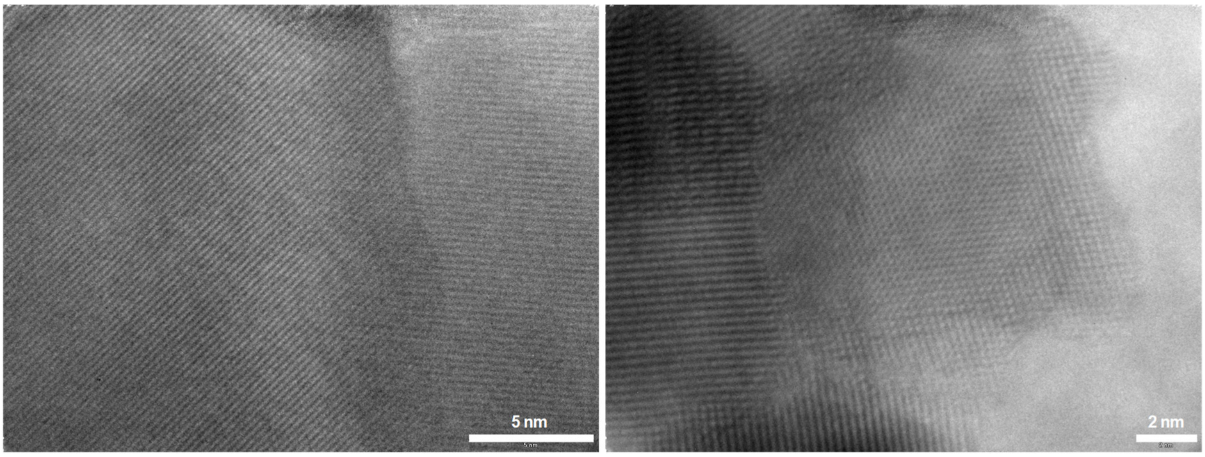

Figure S5. High-resolution TEM images of the crystalline ZnO_{-Me} (Left) and ZnO_{-iPr} (Right) nanoparticles obtained by direct calcinations of $Zn(OMe)_2$ and $Zn(OiPr)_2$.
